# Biodegradable vs. conventional toothbrushes for biofilm control: a systematic review and meta-analysis of randomized trials

**DOI:** 10.3389/froh.2026.1846306

**Published:** 2026-06-24

**Authors:** Frank Mayta-Tovalino, Fran Espinoza-Carhuancho, Cesar Mauricio-Vilchez, Ivan Calderon-Cortez, Miguel Cabanillas-Lazo, Adrian V. Hernandez

**Affiliations:** 1 Unidad de Revisiones Sistemáticas y Meta-análisis (URSIGET), Vicerrectorado de Investigación, Universidad San Ignacio de Loyola, Lima, Peru; 2 Bibliometrics Evidence Evaluation and Systematic Reviews Group (BEERS) Human Medicine Career, Universidad Científica del Sur, Lima, Peru; 3 Research, Innovation and Entrepreneurship Unit, Faculty of Dentistry, Universidad Nacional Federico Villarreal, Lima, Peru; 4 EVIDENTIA Research Group, Universidad Nacional Mayor de San Marcos, Lima, Peru; 5 Neuroscience, Metabolism, Clinical and Healthcare Effectiveness Research Group (NEMECS), Universidad Científica del Sur, Lima, Peru; 6 Health Outcomes, Policy and Evidence Synthesis (HOPES) Group, University of Connecticut School of Pharmacy, Storrs, CT, United States

**Keywords:** biodegradable toothbrushes, dental plaque control, meta-analysis, plastic toothbrushes, systematic review

## Abstract

**Background:**

Due to the immense environmental impact of plastic waste, there has been a significant increase in interest for both biodegradable and natural toothbrushes (BT) as sustainable forms of daily oral care. This includes bamboo toothbrushes, as well as plant-based products (i.e., miswak). As such, these two types of products can be considered similar because they are both renewable and biodegradable in nature. However, their effectiveness when compared to traditional plastic toothbrushes at controlling dental biofilm has not been fully established.

**Objective:**

To evaluate the effectiveness of biodegradable and natural oral hygiene tools compared with conventional plastic toothbrushes for controlling dental plaque in children and adults.

**Methods:**

This systematic review/meta-analysis was reported according to PRISMA guidelines, and its protocol was registered in PROSPERO. All databases were searched using electronic methods from their inception to December 2025: PubMed, Scopus, Web of Science, and Embase. All randomized controlled trials (RCTs) that examined biodegradable/natural oral hygiene products compared to conventional plastic toothbrushes and that had plaque index (PI) as outcome were included. The analyses used random effects model, and certainty of evidence was evaluated using GRADE methodology.

**Results:**

Five RCTs (*n* = 408) met the criteria for inclusion. There was no clear difference in plaque index between biodegradable/natural oral hygiene tools and conventional plastic toothbrushes [mean difference (MD): −0.09, 95% CI: −0.47 to 0.29]. However, the pooled estimate was characterized by very high statistical heterogeneity (*I*^2^ = 89%) and the certainty of the evidence was rated as very low, which substantially limits confidence in this result. The results of subgroup analyses according to type of oral hygiene tool and population did not show any meaningful differences from those of the pooled analysis. The overall certainty of evidence was rated as very low and had a high risk of bias.

**Conclusions:**

Biodegradable and natural oral hygiene products may result in little to no difference in plaque control compared with conventional plastic toothbrushes; however, confidence in this estimate was very low. Well-designed, adequately powered RCTs are required to reduce uncertainty and inform evidence-based clinical and sustainability-related recommendations.

**Systematic Review Registration:**

https://www.crd.york.ac.uk/PROSPERO/view/CRD420251273997, PROSPERO CRD420251273997.

## Introduction

Oral health conditions around the world are an extensive burden to public health. In 2021, an estimated 45,900 out of every 100,000 individuals were living with multiple oral health conditions including untreated decay, severe gum disease, tooth loss and other oral health disorders (1). Also, certain groups of people are affected more than others; for example, it has been documented that the percentage of children with untreated cavities may be as high as 70% depending on the location (2). In addition, approximately 90% of children and adolescents worldwide may experience gingivitis depending on geographic location (3). These statistics highlight the need for effective prevention efforts to be implemented as part of good dental hygiene practices (4).

Toothbrushing has been established as the foundation of good oral hygiene; typically used with mouthwash to supplement each other (5). As toothbrushing occupies such a vital role in the maintenance of good oral health, many recommendations have been published to improve how people brush their teeth and what behaviors are involved in brushing (6). Unfortunately, most of these guidelines were created with minimal certainty of the available evidence, or simply based on expert opinion, which indicates the need for more research on this topic.

Standard toothbrushes have traditionally had handles made of conventional petroleum-based plastic (such as polypropylene) and bristles made of nylon. Conventional plastics are functional and inexpensive but create significant environmental issues due to their long-term persistence in our ecosystem, along with their contribution to the overall build-up of plastic waste. Equally concerning is evidence from the past few years showing that virtually all oral hygiene products produce microplastics during use, and that conventional toothbrushes offer the highest risk for contaminating the environment with microplastics. Contamination with microplastics poses environmental hazards and raises the possibility of microplastic exposure or ingestion by humans through brushing the teeth (1–7).

Environmental issues have encouraged creation and commercialization of biodegradable toothbrushes. The toothbrushes have bamboo handles and various types of acceptable bristle materials, making them a sustainable product because of the speed at which bamboo grows, the ability of bamboo to naturally regenerate, and the relatively lower environmental impact on bamboo production when compared to plastics (8).

Nevertheless, it is unclear whether biodegradable toothbrushes have the capacity to control bacterial plaque; this is a key factor in the prevention of tooth decay and gum disease (9, 10). As the available literature was derived from studies conducted utilizing different methods and sample populations, there is a requirement for a systematic synthesis of this evidence.

Bamboo toothbrushes and miswak were analyzed in the same category of biodegradable/natural oral hygiene tools. However, it is important to understand that there are several key differences between the two tools. The design of the bamboo toothbrush resembles that of a traditional toothbrush; however, bamboo is used instead of plastic. Miswak is a type of traditional chewing stick that requires specific techniques for use and has different types of cultural significance associated with its use. The differences in the way these two types of tools work (mechanism of action) will likely have an impact on clinical outcomes (7, 8).

The aim of the current systematic review and meta-analysis was to compare the efficacy of biodegradable toothbrushes and natural oral hygiene tools with those of conventional toothbrushes in terms of their ability to control bacterial plaque among both children and adults; therefore, findings from the present analysis will provide current evidence to inform clinical decision-making as well as development of public health policy in support of sustainable oral care practices.

## Material and methods

### Study design

This systematic review and meta-analysis were reported in accordance with the Preferred Reporting Items for Systematic Reviews and Meta-Analyses (PRISMA) guidelines (11). The review protocol was registered in the PROSPERO database (Registration Number: CRD420251273997).

### Search strategy

**Figure 1:**
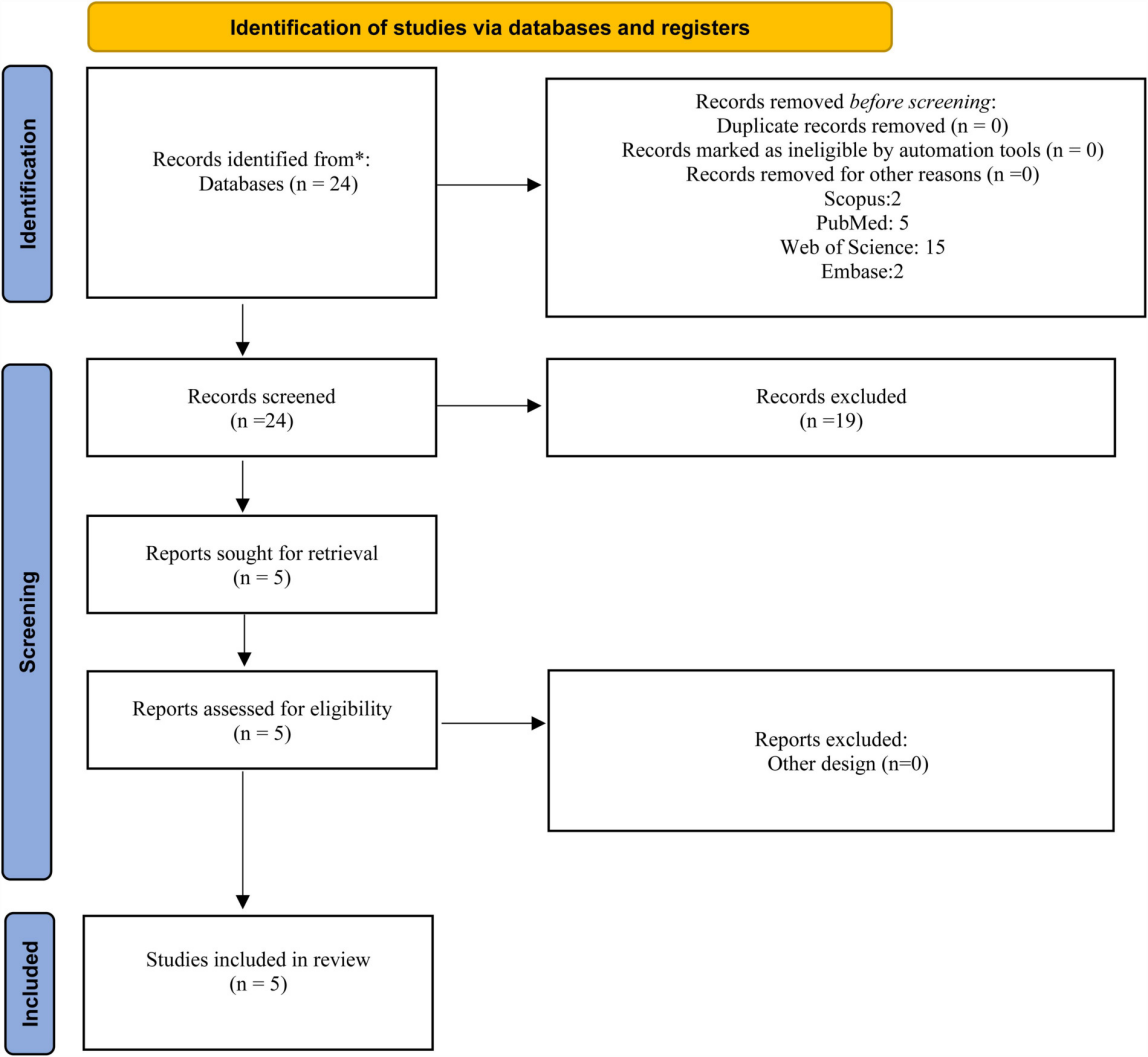
Prisma flow chart.

A comprehensive electronic search was performed in the databases PubMed, Scopus, Web of Science, and Embase from inception to January 7, 2026 (Figure 1). The search strategy combined Medical Subject Headings (MeSH) terms and free-text keywords related to biodegradable toothbrushes and plaque control. The main search terms included: “*biodegradable toothbrush*”, “*bamboo toothbrush*”, “*eco-friendly toothbrush*”, “*plastic toothbrush*”, “*plaque index*”, “*dental plaque*”, “*gingivitis*”, *and* “*randomized controlled trial*”, “*chewing sticks*” *and* “*traditional oral hygiene tools*” (Supplementary Material).

### Eligibility criteria

Randomized controlled trials enrolling participants of any age with natural dentition and without systemic conditions were considered eligible when biodegradable or natural oral hygiene tools—such as bamboo toothbrushes or miswak—were compared with conventional plastic manual toothbrushes, and dental plaque was assessed using validated indices. The concept of “biodegradable toothbrushes” was applied broadly to encompass renewable and environmentally sustainable alternatives, ensuring a comprehensive synthesis of the limited randomized evidence available. No restrictions were imposed regarding language or publication date. Studies were excluded if they were observational in design, assessed interventions not primarily based on mechanical plaque removal, included orthodontic patients, or failed to report plaque-related outcomes. Miswak was included *a priori* given its functional role in mechanical plaque removal and biodegradable nature, thereby strengthening the ecological and clinical relevance of the review.

### Outcome

The plaque index (PI) was the only outcome evaluated in this review. The PI is a numerical value obtained by adding the plaque scores painted on the teeth and dividing it by the total number of surfaces examined. Most studies only reported this outcome.

### Study selection and data extraction

Two reviewers (FEC and FMT) independently selected studies based on eligibility criteria. If there was any disagreement or uncertainty about whether a study met the inclusion criteria, a third reviewer (IC) acted as the final arbiter to make those decisions. The reviewers uploaded all records to Rayyan.ai, using RIS-formatted metadata to help select titles and abstracts. After selecting all eligible studies, data from the studies were extracted using a standardized data extraction form that included the author's name, year of publication, country of the trial, sample size, age range of participants, study design, characteristics of interventions and comparison groups, duration of follow-up, plaque index forms used to assess dental plaque accumulation, and primary outcome measurement.

### Risk of bias assessment

Risk of bias of RCTs were evaluated by two independent reviewers (FEC and FMT) using the Cochrane Risk of Bias 2.0 (ROB 2.0) tool, which assesses potential sources of bias in five domains: randomization process, deviations from intended interventions, missing outcome data, measurement of the outcome, and selection of reported results. The rating for each of the five domains was “low risk”, “some concerns”, or “high risk” according to responses to signaling questions and tool algorithm. Any disagreements between the two reviewers were discussed and resolved between the two reviewers, followed by consultation with a third reviewer, when necessary, until agreement was reached. The overall risk of bias per RCT was low when all domains were at low risk of bias, high when at least one domain was at high risk of bias, and some concerns when at least one domain was at some concerns of risk of bias and there was no domain at high risk of bias.

### Data synthesis and statistical analysis

Meta-analyses were conducted when at least two studies reported comparable quantitative data for the same outcome. As the PI was a continuous outcome measured using the same scale across the included trials, pooled analyses were performed using the mean difference (MD) with corresponding 95% confidence intervals (CI). Given the expected substantial clinical and methodological heterogeneity across RCTs, random-effects models were primarily used. Hartung–Knapp–Siddik–Jonkman adjustment of 95%CIs was used when there were five RCTs or more in an overall meta-analysis or in each stratum of subgroup analyses. Between-study variance (*τ*^2^) was estimated using the restricted maximum likelihood (REML) method. Statistical heterogeneity was assessed using the *I*^2^ statistic (12). Evaluation of small study effects with funnel plots and asymmetry tests was conducted when there were 10 or more RCTs. Subgroup analyses based on toothbrush type (bamboo vs. miswak) and population age (children vs. adults) were also conducted; subgroup effects were significant when the *p* for interaction was <0.10. Certainty of evidence for the evaluated outcome was assessed using the Grading of Recommendations Assessment, Development and Evaluation (GRADE) (13) approach and summarized in a Summary of Findings table.

## Results

### Selection of studies

The systematic electronic search yielded a set of 24 unique records. No records were deemed ineligible prior to the initial screening based upon duplicate publication issues. All records collected for review (*n* = 24) were screened based upon title and abstract. Five records were selected and further underwent full text evaluation; all five studies evaluating 408 individuals were finally selected (8, 14–17). However, it should be noted that some RCTs had more intervention arms than required by our study, therefore there may be a discrepancy between the overall total and the number of subjects analyzed.

### Characteristics of included studies

The five RCTs included 408 subjects from Egypt, Malaysia, Saudi Arabia, and India; all RCTs used subjects from pediatric, adolescent, and adult groups. Three RCTs (15–17) compared miswak with manual toothbrushes in adolescents and adults, while two RCTs (8, 14) [AH3] [FM4] evaluated biodegradable toothbrushes in children (Table 1). [AH5] [FM6] The follow-up period was heterogeneous across RCTs (ranging from immediately after brushing to 12 weeks). The PI was consistently reported in all RCTs reviewed. However, only one RCT (17) reported numerical data for gingival index (GI) outcome; another study (16) reported data for Periodontal Inflamed Surface Area (PISA), and another RCT (15) reported Bleeding index (BI) (%), so they could not be meta-analyzed. On the other hand, biodegradable and natural toothbrushes (e.g., bamboo and neem) produced plaque control results equal to or better than conventional plastic toothbrushes in some cases. Miswak successfully removed plaque, but there were some less than optimal gingival responses depending on how well the technique was used.

**Table 1:** Characteristics of included randomized controlled trials.

Study (Year)	Country	Sample size (n)	Age	Inclusion criteria	Main procedure	Study design	Interventions	Comparison	Follow-up	Main outcomes	Main result	Main conclusion
Ranwa 2025 (8)	India	150	Children 8–12 yrs	Healthy schoolchildren without orthodontic appliances	OHI, parental supervision, random assignment to brush type	Parallel-group RCT	Bamboo and neem toothbrushes	Conventional toothbrush	21 days	PI	Plaque scores showed a significant reduction over time in all three groups. In Group A, mean values decreased from 1.55 ± 0.51 preoperatively to 0.66 ± 0.44 at 1 month and 0.55 ± 0.40 at 3 months. Group B demonstrated a similar decline, from 1.63 ± 0.44 to 0.55 ± 0.60 and 0.48 ± 0.50, respectively. Group C also showed marked improvement, with plaque scores decreasing from 0.92 ± 0.59 to 0.39 ± 0.44 and 0.30 ± 0.40. Overall reductions were statistically significant (*p* = 0.001).	Natural toothbrushes are effective alternatives to conventional plastic brushes
Kariya 2024 (14)	India	90	Children 8–10 yrs	Caries-free children with good general health	Disclosing, supervised brushing with assigned toothbrush, immediate assessment	Randomized controlled trial	Biodegradable toothbrush	Plastic toothbrush	Immediate/same-day	PI	The mean plaque score in Group A was 3.33 ± 0.67 at baseline (day 1), 3.26 ± 0.61 at day 7, and 3.17 ± 0.62 at day 14. In Group B, mean plaque scores were 3.52 ± 0.61 at baseline, 3.28 ± 0.50 at day 7, and 2.77 ± 0.50 at day 14. The mean plaque score in Group C was 3.57 ± 0.54 at baseline, 3.45 ± 0.44 at day 7, and 3.35 ± 0.63 at day 14.	Biodegradable toothbrush is as effective as conventional toothbrush for immediate plaque removal
Fageeh 2024 (15)	Saudi Arabia	30	Adults 20–50 yrs	Systemically healthy participants with gingivitis	Baseline oral examination, OHI, group allocation	Parallel-group RCT	Miswak only	Toothbrush only	4 weeks	PI, Bleeding index (%)	At baseline (T0), mean plaque index values were comparable across groups (detachable miswak: 1.36 ± 0.72; regular toothbrush: 1.37 ± 0.46; bamboo toothbrush: 1.15 ± 0.38). All groups showed progressive reductions in plaque index at 4 weeks (0.84 ± 0.52; 0.92 ± 0.62; 0.82 ± 0.44, respectively) and at T2 (0.66 ± 0.46; 0.60 ± 0.44; 0.50 ± 0.31), with no statistically significant differences between groups at any time point.	Toothbrushing remains superior for gingival health, though miswak Improves plaque control
Azizan 2023 (16)	Malaysia	78	Adolescents 15–17 yrs	Schoolchildren with mild–moderate gingivitis, no orthodontic appliances	OHI session, random group allocation, supervised use	Parallel-group RCT	Miswak	Toothbrush	12 weeks	PI, Periodontal Inflamed Surface Area (PISA)	The MCS group demonstrated a significantly greater improvement in mean PISA values of the anterior teeth compared with the MTB and STB groups (MCS: 16.35 ± 10.03 to 3.41 ± 1.14; MTB: 25.20 ± 14.01 to 3.57 ± 1.19; STB: 26.54 ± 8.64 to 6.17 ± 0.86; *p* < 0.05).	Miswak is as effective as toothbrush in controlling plaque and gingivitis
Abdellatif 2024 (17)	Egypt	60	Adults 18–40 yrs	Healthy adults with gingivitis, no periodontal therapy or antibiotics in last 3 months	Baseline scaling, oral hygiene instruction, random allocation to miswak or toothbrush	Parallel-group RCT	Miswak twice daily	Toothbrush + fluoridated toothpaste	4 weeks	PI, GI (Gingival index)	The miswak group showed no statistically significant change in plaque scores (*p* = 0.58), whereas the toothbrush group demonstrated a significant reduction in plaque levels (*p* = 0.007). Conversely, a significant increase in gingival scores was observed in the miswak group (*p* < 0.001), while no significant change was detected in the toothbrush group (*p* = 0.52).	Miswak is effective for plaque removal but may increase gingival inflammation if technique is improper

S. persica toothbrush (MTB); S. persica chewing stick (MCS); standard tooth-brush (STB).

### Risk of bias assessment

**Figure 2:**
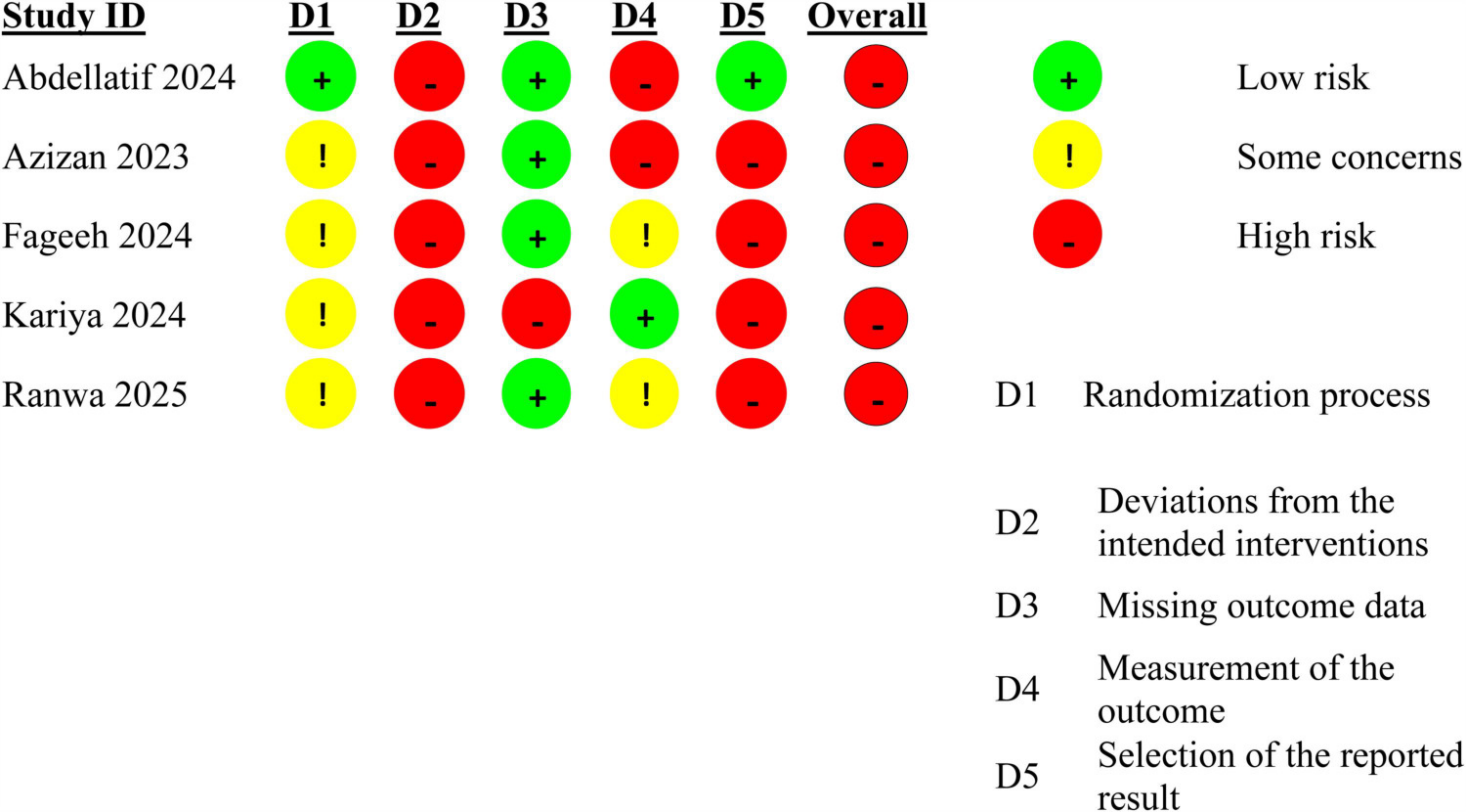
Risk of bias assessment.

All five RCTs were at high risk of bias. Major issues were identified in the domains of randomization process and deviations from intended interventions across all five RCTs. Additional issues were observed in the domains of measurement of the outcome, and selection of reported results (Figure 2).

### Effects of biodegradable and conventional plastic toothbrushes on PI

**Figure 3:**
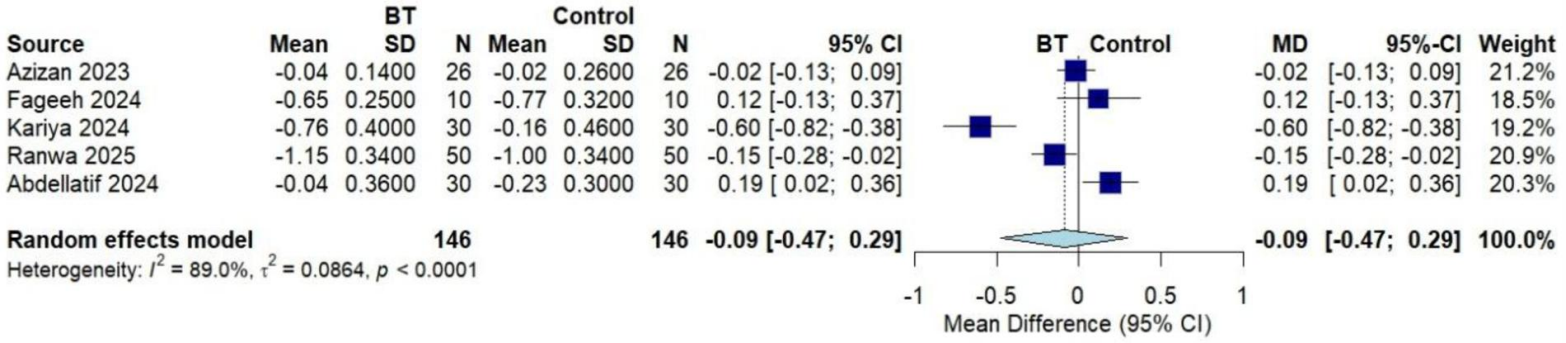
Effects of biodegradable and natural (BT) versus conventional plastic toothbrushes (control) on PI.

Evidence from five RCTs (*n* = 292) indicated no difference in PI between biodegradable/natural oral hygiene tools and conventional plastic toothbrushes (MD: −0.09 units, 95% CI: −0.47 to 0.29, *I*^2^ = 89%; certainty of evidence: very low) (Figure 3 and Table 2).

**Table 2:** Summary of findings table showing certainty of evidence of effects on PI.

Outcomes	Anticipated absolute effects* (95% CI)		№ of participants (studies)	Certainty of the evidence (GRADE)
	Risk with Conventional Toothbrush	Risk with Biodegradable Toothbrush		
Plaque Index (PI) follow-up: range 4 weeks to 12 weeks	The mean Plaque Index was **−0.44** Units	MD **0.09 Units lower** (0.47 lower to 0.29 higher)	292 (5 RCTs)	⊕○○○ Very low^a^^,^^b^^,^^c^
*The risk in the intervention group (and its 95% confidence interval) is based on the assumed risk in the comparison group and the **relative effect** of the intervention (and its 95% CI). **CI:** confidence interval; **MD:** mean difference				
**GRADE Working Group grades of evidence** **High certainty:** we are very confident that the true effect lies close to that of the estimate of the effect. **Moderate certainty:** we are moderately confident in the effect estimate: the true effect is likely to be close to the estimate of the effect, but there is a possibility that it is substantially different. **Low certainty:** our confidence in the effect estimate is limited: the true effect may be substantially different from the estimate of the effect. **Very low certainty:** we have very little confidence in the effect estimate: the true effect is likely to be substantially different from the estimate of effect.				

Explanations.

a All studies presented a high risk of bias according to the Rob2.0 tool.

b The i2 measuring heterogeneity was 89%.

c Studies evaluating children and adults were compared due to the scarcity of published RCTs on the subject.

### Subgroup analyses

**Figure 4:**
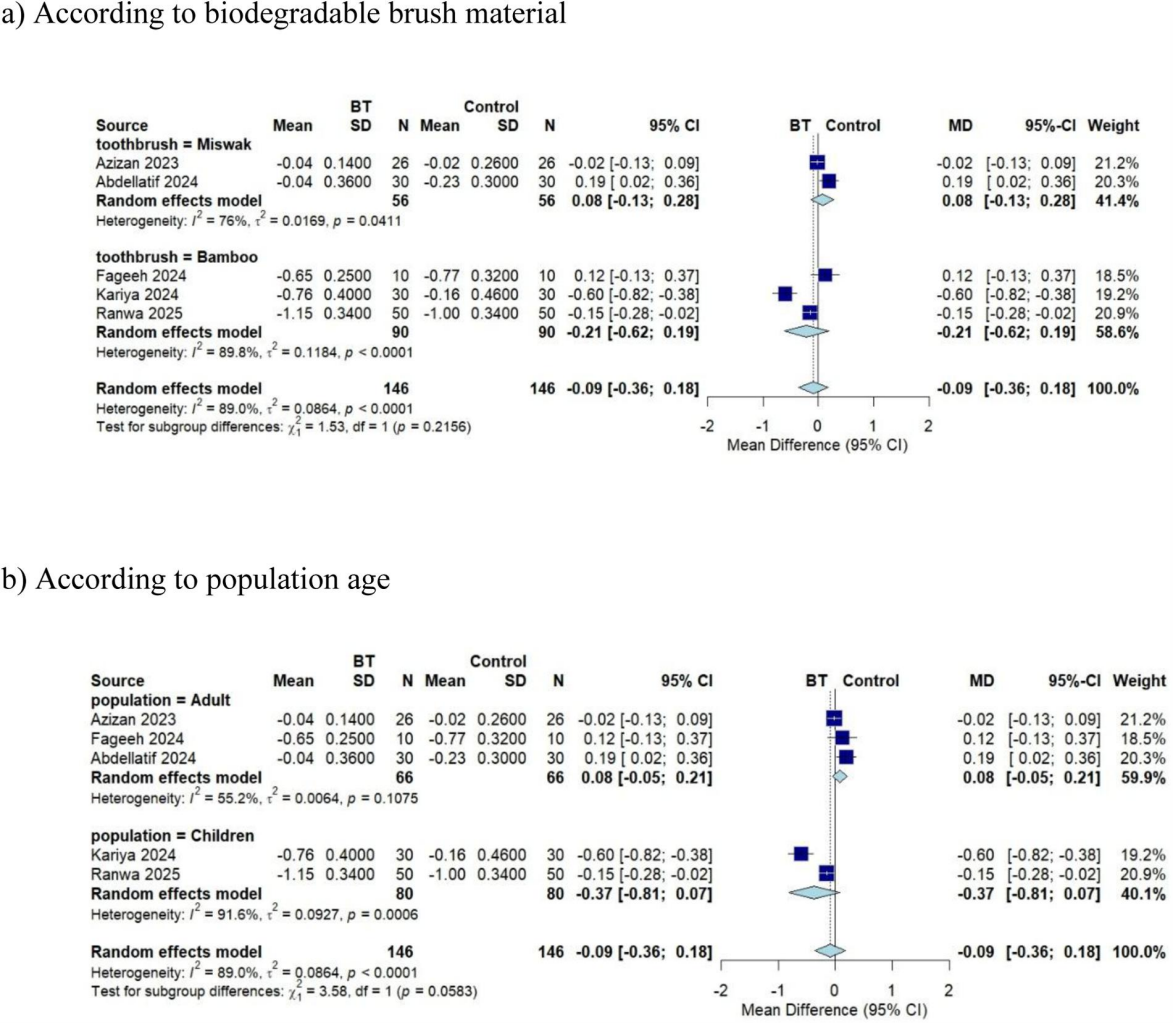
Subgroup analyses of the effects of biodegradable and natural (BT) versus conventional plastic toothbrushes (control) on PI. **(a)** According to biodegradable brush material. **(b)** According to population age.

Subgroup analysis according to the type of biodegradable toothbrush (bamboo vs. miswak) showed no statistically significant difference in plaque index (PI) between biodegradable and conventional plastic toothbrushes (*p* for interaction = 0.21, Figure 4a). Subgroup analysis according to population age (adults vs. children) showed significant subgroup effects (*p* for interaction = 0.05). However, In the adult population (*n* = 146), the pooled analysis showed no difference in PI between biodegradable and conventional plastic toothbrushes (MD: −0.09, 95% CI: −0.36 to 0.18, *I*^2^ = 89%) (Figure 4b). Given the small number of studies and the borderline statistical significance, these subgroup findings should be interpreted with caution and considered exploratory rather than confirmatory.

## Discussion

The decision to categorize bamboo toothbrushes, miswak and other natural oral hygiene implements as “biodegradable/natural toothbrushes” was intentional and made prior to the start of the study, in order to ensure that the study was as comprehensive as possible, due to the limited availability of randomized data. All of these types of oral hygiene products have different modes of action, different methods of use, and different cultural contexts. Howeverthey all share similarities, in that they are composed of renewable and biodegradable materials, and are intended to be viewed as an environmentally sustainable alternative to using conventional plastic toothbrushes.

This systematic review with meta-analysis, which included five RCTs with a sample size of 408 participants, found that the use of biodegradable toothbrushes had no statistically significant effect, considering subgroups according to biodegradable material and population type, on PI, with very low certainty of evidence.

Although plaque-related outcomes were able to be quantitatively combined, there are several other clinically relevant periodontal indices that were assessed in the included studies [Bleeding Index (%), Periodontal Inflamed Surface Area (PISA), and Gingival Index] but cannot be quantitatively combined because they were only reported in one study per index. Therefore, as these types of results cannot be included in a meta-analysis, it limits any definitive conclusions on the effects of biodegradable toothbrushes on gingival inflammation and periodontal status. Therefore, the available body of evidence does not adequately provide information to support whether biodegradable toothbrushes improve periodontal health in addition to plaque control.

Our results indicate that there is no significant difference between biodegradable and conventional toothbrushes in terms of plaque control. Importantly, the observed pooled mean difference (MD = −0.09) is likely below the minimal clinically important difference for commonly used plaque indices, suggesting that even if statistical significance had been reached, the clinical relevance of such a difference would be questionable. The substantial heterogeneity observed (*I*^2^ = 89%) likely reflects genuine clinical and methodological diversity rather than random variation. Differences in participant age, baseline oral health status, brushing supervision, follow-up duration, co-interventions such as scaling or oral hygiene instruction, and the intrinsic characteristics of natural oral hygiene tools (e.g., miswak vs. bamboo toothbrushes) may have contributed significantly to the variability in effect estimates.

Consistent with our results, the study by Sharma et al. (18) showed no significant differences in plaque removal capacity between chewing brushes and manual toothbrushes. Their meta-analysis showed that the overall combined effect was not significant (MD = −0.02; 95% CI: −0.29 to 0.24; *Z* = 0.17; *p* = 0.86), despite considerable heterogeneity between these two studies (*I*^2^ = 89%). Both proved to be effective in temporarily removing plaque, with neither being superior to the other in most trials. These results support the interpretation that chewing toothbrushes do not offer any advantage over conventional toothbrushes and should only be recommended in very specific contexts, such as for children or people with disabilities or reduced manual dexterity, as our results suggest (18).

Similarly, a systematic review conducted by Hoogteijling et al. (19) evaluating the effectiveness of chewable toothbrushes vs. conventional toothbrushes on the plaque index covered 10 studies that included chewable toothbrushes made of biodegradable polymers supplemented with xylitol. The meta-analysis, which included four RCTs with moderate and high risk of bias, mainly due to problems with blinding of evaluators, showed no statistically significant association (MD: 0.06; 95% CI: −0.26 to 0.37; *I*^2^ = 87%) without assessment of the certainty of the evidence. Another meta-analysis comparing the efficacy of tapered toothbrush filaments compared to end-rounded filaments with seven RCTs found no statistically significant difference for dental plaque index (MD: 0.07; 95% CI: −0.18 to 0.32; *I*^2^ = 89%) with low certainty of evidence due to inconsistency and imprecision (19).

In contrast, a meta-analysis conducted by Dağdeviren et al., evaluating the effectiveness of power toothbrushes vs. manual toothbrushes determined that, with moderate certainty of evidence, power toothbrushes have a small but statistically significant effect on the plaque index (MD: −0.22; 95% CI: −0.36 to −0.07; *I*^2^ = 0%) (20). This pattern of results could be explained by the fact that modifications to toothbrushes that do not favor the biomechanics of plaque removal, but only modify the toothbrush material, may not have a significant clinical impact. This is also supported by a systematic review that evaluated orthodontic brushes compared to traditional brushes, where the former statistically and significantly reduced the plaque index (MD: −1.72; 95% CI: −2.61 to −0.83; *I*^2^ = 82%) (21). Similar to our findings, these results suggest that biomechanical design features are more critical determinants of plaque control efficacy than material composition alone.

Although our findings reveal no statistically significant differences between biodegradable toothbrushes and traditional toothbrushes on a plaque index, one should make their choice based on other factors, including long-term sustainability and environmental considerations. Life cycle analyses indicate that using a bamboo toothbrush will generate 11 times less greenhouse gas emissions than using an electric toothbrush and require 97 percent less virgin plastic to produce than a traditional manual toothbrush. Additionally, in order to evaluate true sustainability, one needs to consider the social aspects of a product's life cycle. Recent studies have shown that although bamboo toothbrushes do have an environmental advantage, their production through globalized supply chains has inherent high levels of social risk (the social dimension of sustainability), i.e., workplace conditions, fair wages, and governance in the production country. In contrast, circular economy models utilizing renewable energy and recycling represent the best way of achieving an optimal blend of minimizing carbon footprint and reducing social harm. Therefore, although both types of toothbrushes are identical in terms of clinical efficacy, recommendations on toothbrushes should be made using the principles of multidimensional sustainability, optimally addressing environmental, social, and public health issues simultaneously. It is also essential to interpret subgroup analyses cautiously because of their limited number of studies and participants and therefore likely underpowered to identify meaningful differences (22, 23).

The certainty of evidence was rated as very low due to high risk of bias, substantial heterogeneity, and indirectness. The high risk of bias was mainly due to problems related to the blinding process, intervention allocation, and selective reporting of results, so we recommend that future clinical trials follow the CONSORT guidelines (24). The substantial heterogeneity observed could not be explained solely by the brush material or patient age, suggesting that additional factors such as brushing technique, duration, frequency of use, baseline oral health status, patient adherence, and baseline clinical conditions may contribute significantly to the variability in results. We therefore recommend that future RCTs systematically evaluate and report these variables. Furthermore, since most of the included studies recruited African and Asian populations, we recommend conducting studies in other populations, including those in the Americas and Europe, as ethnic differences in the composition of the oral microbiome have been documented and may influence treatment response (25), which would improve the generalizability of the findings in different cultural and epidemiological contexts. Finally, given that our findings and evidence from previous reviews suggest that material modification alone does not significantly impact plaque control, we recommend that future RCTs focus on interventions that alter the biomechanical function of brushing, such as bristle configuration, head design, or handle ergonomics, rather than solely material substitution.

Interpretation of our results must consider the limitations of our systematic review and meta-analysis. One limitation is that we did not include gray literature. This means that any RCT published in a non-indexed journal or an unpublished study was likely excluded from our review and may have contributed to potential gaps in our evidence base as well as introducing publication bias. The second limitation is that we did not perform an analysis of observational studies. Observational studies may supply useful information for determining treatment effectiveness in actual practice settings, while RCT are considered to provide the best available evidence for treatment efficacy. The longest follow-up duration from the included study was only 12 weeks. Thus, the data available to make conclusions on the long-term benefits of biodegradable toothbrushes on plaque control are insufficient to support strong claims regarding the long-term use of biodegradable toothbrushes for patients. Another limitation is that we did not evaluate the impact of toothbrushes on patients' satisfaction and/or preferences on their use, which affects the likelihood of their use and may impact the effectiveness of the toothbrush. As such, based on GRADE's risk of bias assessment, all the studies included were deemed to bear a high risk of bias mainly based on inadequate reporting of the randomization process, lack of blinding, and potential selective reporting (26). These limitations reduce confidence in pooled estimates and yield a low level of certainty rating based on GRADE.

The methodological strength of this systematic review greatly increases the validity and trustworthiness of this study's conclusions. First, evaluating the certainty of the evidence through the GRADE framework permits a clear way to evaluate the degree of confidence in the study's findings and to support the use of evidence-based practice in clinical decisions. Second, by not limiting our search (i.e., to exclude searches by date of publication or language), we have reduced the impact of language bias and ensured comprehensive access to available literature. Third, we used the PRISMA guideline to report all sections of our systematic review. Fourth, registering the protocol in PROSPERO before the commencement of this systematic review increases transparency and reduces the potential for selective reporting of outcomes. Finally, random effects modelling was the most appropriate method for conducting the meta-analysis due to the high levels of clinical and statistical variability between the studies included in this systematic review; therefore, random effects modelling provides a more conservative and realistic estimate of treatment effects. Finally, among the limitations of this review are the very limited number of randomized clinical trials (*n* = 5), this indicates that there is very limited evidence available to support the findings of this review; as a result of this, the strength of the pooled estimates and the ability to assess variability of the results is reduced, thus the certainty of the conclusions from this review are decreased. The findings from this review should therefore be regarded as preliminary, requiring further well-designed, adequately powered randomised clinical trials to enhance the quality of evidence and to offer better quality guidance for clinical practice and sustainability.

The limitation of this review is that only the plaque index was available as a primary outcome that could be pooled from the studies evaluated in this meta-analysis. There were two studies that reported both gingival index and bleeding index results, but because both indices measure different constructs, we could not pool these for analysis. Likewise, other potentially useful metrics were not consistently examined across all studies (for example, PISA). Ultimately, the limited availability of data for calculating other outcomes means that our data analysis only included the plaque index. Because our analysis did not include any other clinically relevant outcomes or types of analyses, we highlight the need for future clinical trials to utilize standardized and thorough methods for measuring outcomes.

The findings of the meta-analysis showed an extremely large degree of statistical heterogeneity (*I*^2^ = 89%) making it impossible to rely on the estimated pooled result. This variability is not indicative of a problem with the review methodology, but rather represents a true difference in the characteristics of the studies included in the analysis, such as the follow-up duration, supervision of tooth brushing, initial oral health state and substantial variance between the experimental and control groups. Due to the limited number of studies, the uniformly high risk of bias inherent to these studies precluded the use of meta-regression or sensitivity analyses. However, this heterogeneity presents a significant opportunity for researchers to avoid errors and misinterpretation, and indicates a need for further trials with the same outcome measures, longer follow-up, and stricter methodological and methodological standards to minimize uncertainty and increase the strength of the evidence base.

## Conclusions

Biodegradable or natural toothbrushes may have little or no effect on oral health compared to conventional plastic toothbrushes in children and adults; however, the evidence is very uncertain. Subgroup analyses of the two types of toothbrushes (bamboo and miswak) and the two populations (children and adults) showed no significant differences between the two groups. The certainty of the evidence associated with these findings was rated as “very low” according to the GRADE system due to significant heterogeneity, imprecision, and high risk of bias in the included randomized controlled trials. These findings indicate that biodegradable toothbrushes may represent an option for daily oral hygiene; however, we recommend caution when interpreting these results. Future research should include well-designed, adequately powered randomized controlled trials with standardized outcome measures, appropriate blinding, and longer follow-up periods than are currently required to reduce uncertainty and enable well-founded clinical and public health recommendations. The findings from our review suggest that all randomized trials included in this analysis had a high risk of bias and that pooling the results from all of the studies resulted in considerable heterogeneity and imprecision, which limits the ability to generalize the findings.

## Data Availability

The original contributions presented in the study are included in the article/Supplementary Material, further inquiries can be directed to the corresponding author.

## References

[1] GBD 2021 Oral Disorders Collaborators. Trends in the global, regional, and national burden of oral conditions from 1990 to 2021: a systematic analysis for the global burden of disease study 2021. Lancet. (2025) 405(10482):897–910. 10.1016/S0140-6736(24)02811-3 40024264

[2] Abutayyem H Alam MK Al Shayeb M Hashim R. A systematic review and meta-analysis of the prevalence of dental caries in the permanent teeth of Arab children. Eur J Dent. (2025) 19(2):275–85. 10.1055/s-0044-1795117 39750524 PMC12020594

[3] Elgasmi FE Maghous K Badre B. Gingivitis in children and adolescents: epidemiological overview and associated factors-A narrative review. Front Oral Health. (2025) 6:1675033. 10.3389/froh.2025.1675033 41140336 PMC12549658

[4] Fan W Liu C Zhang Y Yang Z Li J Huang S. Epidemiology and associated factors of gingivitis in adolescents in Guangdong Province, Southern China: a cross-sectional study. BMC Oral Health. (2021) 21(1):311. 10.1186/s12903-021-01666-1 34134691 PMC8207589

[5] Gallione C Bassi E Cattaneo A Busca E Basso I Dal Molin A. Oral health care: a systematic review of clinical practice guidelines. Nurs Health Sci. (2025) 27(1):e70027. 10.1111/nhs.70027 39776243 PMC11707404

[6] Glenny AM Walsh T Iwasaki M Kateeb E Braga MM Riley P. Development of tooth brushing recommendations through professional consensus. Int Dent J. (2024) 74(3):526–35. 10.1016/j.identj.2023.10.018 38052700 PMC11123540

[7] Protyusha GB Kavitha B Robin RS Nithin A Ineyathendral TR Shivani SS. Microplastics in oral healthcare products (OHPs) and their environmental health risks and mitigation measures. Environ Pollut. (2024) 343:123118. 10.1016/j.envpol.2023.123118 38092338

[8] Ranwa S Manohar B Patel R Shekhawat D. A comparative study on the efficacy of plaque removal using conventional toothbrushes, bamboo toothbrushes, and neem toothbrushes among school-going children. Int J Clin Pediatr Dent. (2025) 18(4):420–4. 10.5005/jp-journals-10005-3111 40469829 PMC12131066

[9] Khalaf MS Qasim AA Jafar ZJ Mohammad AT. Dental plaque caries related microorganism in relation to demographic factors among a group of Iraqi children. Folia Med (Plovdiv). (2024) 66(4):491–9. 10.3897/folmed.66.e127454 39257269

[10] Newman HN. Plaque and chronic inflammatory periodontal disease. A question of ecology. J Clin Periodontol. (1990) 17(8):533–41. 10.1111/j.1600-051X.1990.tb01102.x 2212083

[11] Page MJ McKenzie JE Bossuyt PM Boutron I Hoffmann TC Mulrow CD. The PRISMA 2020 statement: an updated guideline for reporting systematic reviews. Syst Rev. (2021) 10(1):89. 10.1186/s13643-021-01626-4 33781348 PMC8008539

[12] Thorlund K Imberger G Johnston BC Walsh M Awad T Thabane L. Evolution of heterogeneity (*I*^2^) estimates and their 95% confidence intervals in large meta-analyses. PLoS One. (2012) 7(7):e39471. 10.1371/journal.pone.0039471 22848355 PMC3405079

[13] Brozek JL Canelo-Aybar C Akl EA Bowen JM Bucher J Chiu WA. GRADE guidelines 30: the GRADE approach to assessing the certainty of modeled evidence-an overview in the context of health decision-making. J Clin Epidemiol. (2021) 129:138–50. 10.1016/j.jclinepi.2020.09.018 32980429 PMC8514123

[14] Kariya PB Desai A Singh S Bansal B Shah Y. Comparing plaque removal efficacy of biodegradable toothbrush and nonbiodegradable toothbrush in children of 8–10 years of age: a randomized clinical study. J Indian Soc Pedod Prev Dent. (2024) 42(2):112–8. 10.4103/jisppd.jisppd_61_24 38957908

[15] Fageeh HN Mansoor MA Fageeh HI Abdul HN Mawkili LK Faris SM. Plaque removal and gingival bleeding using biodegradable toothbrushes: salvadora persica, bamboo, and nylon: a comparative study. Med Sci Monit. (2024) 30:e944469. 10.12659/MSM.944469 39511862 PMC11555888

[16] Azizan NF Mohd N Nik Azis NM Baharin B. Effectiveness of Salvadora persica toothbrush and Salvadora persica chewing stick in plaque and gingivitis control: a randomized control trial. BMC Complement Med Ther. (2023) 23(1):456. 10.1186/s12906-023-04295-z 38098022 PMC10720088

[17] Abdellatif HM Hebbal M Alsagob E Alsaleh A Mwena A Almusaad M. Comparative effectiveness of miswak and toothbrushing on dental plaque and gingivitis: a randomized controlled trial. Healthcare (Basel). (2024) 12(21):2150. 10.3390/healthcare12212150 39517363 PMC11545077

[18] Sharma H Ruikar M. Effectiveness of chewable toothbrushes compared to manual toothbrushes in removing dental plaque—a systematic review and meta-analysis. Indian J Dent Res. (2022) 33(4):445–51. 10.4103/ijdr.ijdr_1158_21 37006013

[19] Hoogteijling F Hennequin-Hoenderdos NL Van der Weijden GA Slot DE. The effect of tapered toothbrush filaments compared to end-rounded filaments on dental plaque, gingivitis and gingival abrasion: a systematic review and meta-analysis. Int J Dent Hyg. (2018) 16(1):3–12. 10.1111/idh.12272 28173609

[20] Dağdeviren F Van der Weijden GA Zijlstra CP Slot DE. The effectiveness of power versus manual toothbrushes on plaque removal and gingival health in children—a systematic review and meta-analysis. Int J Dent Hyg. (2025) 23(4):682–702. 10.1111/idh.12915 40739767 PMC12516003

[21] Marçal FF Mota de Paulo JP Barreto LG de Carvalho Guerra LM Silva PGB. Effectiveness of orthodontic toothbrush versus conventional toothbrush on plaque and gingival index reduction: a systematic review and meta-analysis. Int J Dent Hyg. (2022) 20(1):87–99. 10.1111/idh.12511 33971076

[22] Lyne A Ashley P Saget S Porto Costa M Underwood B Duane B. Combining evidence-based healthcare with environmental sustainability: using the toothbrush as a model. Br Dent J. (2020) 229(5):303–9. 10.1038/s41415-020-1981-0 32918023

[23] Brett D Ingeborg S Rodrigo M Paul A. What is the best toothbrush from a social impact perspective? J Dent. (2025) 156:105708. 10.1016/j.jdent.2025.105708 40127750

[24] Schulz KF Altman DG Moher D, CONSORT Group. CONSORT 2010 statement: updated guidelines for reporting parallel group randomised trials. BMJ (2010) 340:c332. 10.1136/bmj.c332 20332509 PMC2844940

[25] Premaraj TS Vella R Chung J Lin Q Hunter P Underwood K. Ethnic variation of oral microbiota in children. Sci Rep. (2020) 10(1):14788. 10.1038/s41598-020-71422-y 32901068 PMC7478955

[26] Dettori JR Norvell DC Chapman JR. Fixed-effect vs random-effects models for meta-analysis: 3 points to consider. Global Spine J. (2022) 12(7):1624–6. 10.1177/21925682221110527 35723546 PMC9393987

