# Supplementary material for: Biodegradable vs. conventional toothbrushes for biofilm control: a systematic review and meta-analysis of randomized trials
Source: Front Oral Health. 2026 Jun 24;7:1846306. doi: 10.3389/froh.2026.1846306 (PMC13341797; doi:10.3389/froh.2026.1846306)
Supplement: Supplementary file 1 [file Table1.docx]

**Biodegradable products vs. conventional toothbrushes in controlling biofilm in children and/or adults: A meta-analysis**

**Supplementary material S1**

**Search strategies**

| **Engine** | **Strategy** | **Results** |
| --- | --- | --- |
| **Pubmed** | ( "biodegradable toothbrush*" OR "compostable toothbrush*" OR "sustainable toothbrush*" OR "eco toothbrush*" OR "green toothbrush*" OR "zero-waste toothbrush*" OR "plastic-free toothbrush*" OR "bamboo-based toothbrush*" OR "wooden-based toothbrush*" OR "plant-based toothbrush*" OR "natural-fiber toothbrush*" OR "renewable material toothbrush*" OR "sustainable oral care product*" OR "eco-friendly oral hygiene device*" ) | **5** |
|  | ( "plastic toothbrush*" OR "petroleum-based toothbrush*" OR "polypropylene toothbrush*" OR "nylon-bristle toothbrush*" OR "synthetic toothbrush*" OR "conventional toothbrush*" OR "disposable toothbrush*" OR "traditional toothbrush*" OR "non-biodegradable toothbrush*" OR "non-compostable toothbrush*" OR "standard toothbrush*" OR "single-use toothbrush*" ) |  |
|  | ("Randomized Controlled Trial" OR "Clinical Trials, Randomized" OR "Trials, Randomized Clinical" OR "Controlled Clinical Trials, Randomized") |  |
| **Scopus** | TITLE-ABS-KEY( "biodegradable toothbrush*" OR "compostable toothbrush*" OR "sustainable toothbrush*" OR "eco toothbrush*" OR "green toothbrush*" OR "zero-waste toothbrush*" OR "plastic-free toothbrush*" OR "bamboo-based toothbrush*" OR "wooden-based toothbrush*" OR "plant-based toothbrush*" OR "natural-fiber toothbrush*" OR "renewable material toothbrush*" OR "sustainable oral care product*" OR "eco-friendly oral hygiene device*" ) | **2** |
|  | TITLE-ABS-KEY ( "plastic toothbrush*" OR "petroleum-based toothbrush*" OR "polypropylene toothbrush*" OR "nylon-bristle toothbrush*" OR "synthetic toothbrush*" OR "conventional toothbrush*" OR "disposable toothbrush*" OR "traditional toothbrush*" OR "non-biodegradable toothbrush*" OR "non-compostable toothbrush*" OR "standard toothbrush*" OR "single-use toothbrush*" ) |  |
|  | TITLE-ABS-KEY ("Randomized Controlled Trial" OR "Clinical Trials, Randomized" OR "Trials, Randomized Clinical" OR "Controlled Clinical Trials, Randomized") |  |
| **Web of Science** | ( "biodegradable toothbrush*" OR "compostable toothbrush*" OR "sustainable toothbrush*" OR "eco toothbrush*" OR "green toothbrush*" OR "zero-waste toothbrush*" OR "plastic-free toothbrush*" OR "bamboo-based toothbrush*" OR "wooden-based toothbrush*" OR "plant-based toothbrush*" OR "natural-fiber toothbrush*" OR "renewable material toothbrush*" OR "sustainable oral care product*" OR "eco-friendly oral hygiene device*" ) | **15** |
|  | ( "plastic toothbrush*" OR "petroleum-based toothbrush*" OR "polypropylene toothbrush*" OR "nylon-bristle toothbrush*" OR "synthetic toothbrush*" OR "conventional toothbrush*" OR "disposable toothbrush*" OR "traditional toothbrush*" OR "non-biodegradable toothbrush*" OR "non-compostable toothbrush*" OR "standard toothbrush*" OR "single-use toothbrush*" ) |  |
|  | ("Randomized Controlled Trial" OR "Clinical Trials, Randomized" OR "Trials, Randomized Clinical" OR "Controlled Clinical Trials, Randomized") |  |
| **Embase** | ( "biodegradable toothbrush*" OR "compostable toothbrush*" OR "sustainable toothbrush*" OR "eco toothbrush*" OR "green toothbrush*" OR "zero-waste toothbrush*" OR "plastic-free toothbrush*" OR "bamboo-based toothbrush*" OR "wooden-based toothbrush*" OR "plant-based toothbrush*" OR "natural-fiber toothbrush*" OR "renewable material toothbrush*" OR "sustainable oral care product*" OR "eco-friendly oral hygiene device*" ) | **2** |
|  | ( "plastic toothbrush*" OR "petroleum-based toothbrush*" OR "polypropylene toothbrush*" OR "nylon-bristle toothbrush*" OR "synthetic toothbrush*" OR "conventional toothbrush*" OR "disposable toothbrush*" OR "traditional toothbrush*" OR "non-biodegradable toothbrush*" OR "non-compostable toothbrush*" OR "standard toothbrush*" OR "single-use toothbrush*" ) |  |
|  | ("Randomized Controlled Trial" OR "Clinical Trials, Randomized" OR "Trials, Randomized Clinical" OR "Controlled Clinical Trials, Randomized") |  |

# Supplementary Material S2 PRISMA 2020 Checklist

Title of the review:
Effectiveness of biodegradable and natural oral hygiene product versus conventional plastic toothbrushes in controlling biofilm in children and/or adults: A systematic review and meta-analysis

| Item | Checklist item | Location in manuscript |
| --- | --- | --- |
| 1 | Identify the report as a systematic review and/or meta-analysis | Title page |
| 2 | Structured summary | Abstract |
| 3 | Rationale for the review | Introduction |
| 4 | Objectives | Introduction |
| 5 | Eligibility criteria | Methods – Eligibility criteria |
| 6 | Information sources and search date | Methods – Search strategy |
| 7 | Full search strategy | Supplementary material |
| 8 | Study selection process | Methods – Study selection |
| 9 | Data extraction process | Methods – Data extraction |
| 10 | Outcomes definition | Methods – Outcomes |
| 11 | Risk of bias assessment | Methods – Risk of bias |
| 12 | Effect measures | Methods – Statistical analysis |
| 13 | Synthesis methods | Methods – Statistical analysis |
| 14 | Reporting bias assessment | Methods – Statistical analysis |
| 15 | Certainty of evidence (GRADE) | Methods – GRADE |
| 16 | Study selection results | Results – PRISMA flow diagram |
| 17 | Study characteristics | Results – Table 1 |
| 18 | Risk of bias results | Results – Figure 2 |
| 19 | Individual study results | Results – Table 1 |
| 20 | Synthesis results | Results – Figure 3 |
| 21 | Reporting bias results | Not assessed |
| 22 | Certainty of evidence results | Results – Table 2 |
| 23 | Discussion and interpretation | Discussion |
| 24 | Protocol registration | Methods – PROSPERO |
| 25 | Funding | Funding section |
| 26 | Conflicts of interest | Conflicts of interest |
| 27 | Data availability | Data availability statement |
